# Mitigation of Delamination Between Bilayer Membranes with Low Affinity to Enable Roll-to-Roll Fabrication

**DOI:** 10.3390/membranes16100314

**Published:** 2026-09-25

**Authors:** Minwoo Jung, Eesh M. Kulshrestha, Isabel C. Escobar, Tequila A. L. Harris

**Affiliations:** 1 George W. Woodruff School of Mechanical Engineering, Georgia Institute of Technology, Atlanta, GA 30332, USA; 2 Department of Chemical and Materials Engineering, Stanley and Karen Pigman College of Engineering, University of Kentucky, Lexington, KY 40506, USAisabel.escobar@uky.edu (I.C.E.)

**Keywords:** bilayer membranes, delamination mitigation, scalable membrane fabrication, slot-die coating on a R2R, eco-friendly solvents

## Abstract

Single-layer membranes, despite their ease of fabrication and performance, often face material characteristic limitations that are employed for membrane fabrication, such as polymer fouling propensity and low mechanical strength. To mitigate such material drawbacks, bilayer membranes were developed to use multiple materials with the potential to mitigate the disadvantages of using a single material. When fabricating bilayer membranes with different polymers, varying affinities between the polymers can lead to significant challenges. Specifically, if the affinity between the polymers is low, delamination often occurs. This phenomenon poses a substantial limitation on the combinations of polymers that can be utilized in the construction of bilayer membranes. Understanding and addressing the affinity issues are crucial for membrane performance and expanding the range of applicable materials. This work explores the behavior of two low-affinity polymeric membrane materials: polyvinylidene fluoride-co-hexafluoropropylene (PVDF-HFP) and polysulfone (PSf), which are prone to delamination. PSf and PVDF-HFP were selected due to their relative RED values and their established applications in membrane fabrication. A scalable process incorporating slot-die coating on a roll-to-roll (R2R) system followed by non-solvent-induced phase inversion (NIPS) is used to fabricate the bilayer membranes. To mitigate delamination between these polymers, additive polymers were strategically employed: poly(methyl methacrylate) (PMMA) to enhance interfacial affinity, or polydopamine (PDA) to promote hydrogen bonding. This approach yielded an ultrafiltration bilayer membrane that achieved a 2B rating on the ASTM D3359-B standard for adhesion at the interface between layers. Scaled fabrication of bilayer membranes is enabled by this work.

## 1. Introduction

Single-layer polymeric membranes are extensively utilized due to their relative ease of fabrication and performance. However, these membranes have certain limitations stemming from the use of a single material for the membrane matrix [1]. Some polymers employed in these membranes exhibit low resistance to fouling, chloride ions, and acidic conditions [2,3]. To address these drawbacks, research has focused on composite or bilayer membranes, which allow for properties beyond those of single materials. Incorporating multiple materials into a single-layer composite membrane has been investigated to introduce multifunctionality or improve membrane characteristics [4]. Another approach involves adding additional layers to create bilayer membranes, thereby enhancing functionality and mitigating limitations. The bilayer strategy offers greater flexibility in material selection and functionalization due to the increased number of layers, which broadens the scope for tailoring membrane properties [5,6,7]. Current research continues to explore the pairing of different layers to overcome material limitations and enhance features such as anti-fouling, hydrophilicity, and cost-effectiveness.

To fabricate bilayer membranes, various techniques such as solution casting, grafting, electrospinning, interfacial polymerization, and extrusion are employed [8,9,10,11]. Among these, solution casting is particularly prevalent for scalable production, especially when utilizing equipment such as slot-dies, doctor blades, or spray-coating tools within a roll-to-roll (R2R) system [12,13]. The solution casting process for bilayer membranes involves multiple coating techniques originating from the coating industry. These methods include sequential deposition of solutions on an existing film, layer-by-layer deposition, and simultaneous deposition of all solutions in a single step. Sequential deposition entails repeated casting of dope solutions followed by a phase separation process. The layer-by-layer approach involves repeated casting to create a bilayer dope solution and then a secondary step for solidifying the membrane, phase separation. Here, phase separation is conducted using a non-solvent-induced phase separation (NIPS) approach. The simultaneous deposition method requires only one casting of a bilayer dope solution, followed by a single-phase separation. Regardless of the chosen method, bilayer membrane fabrication via casting requires casting multiple materials and subsequent phase separation steps.

However, casting bilayer dope solutions and fabricating bilayer membranes via phase separation present specific fabrication challenges. When polymeric membranes are produced by simultaneously casting dope solutions into a thin film and inducing phase separation to form a membrane, delamination defects frequently occur if there is low affinity between the different polymer materials or a mismatch in hydrophilicity [14,15,16]. Low-affinity material delamination has also been observed in other fields, such as metal coating, fluid coating, and semiconductors [17]. In polymeric membrane applications, delamination of bilayer structures can compromise membrane integrity and reduce overall lifespan, especially as further delamination may occur under high-pressure operation. Moreover, delaminated membranes can permit lateral water movement, which may impair rejection performance. Consequently, addressing delamination is a crucial aspect of bilayer membrane fabrication. Many studies used grafting or surface treatment techniques to overcome layer delamination; however, these studies required additional steps for membrane fabrication to increase layer adhesion for subsequent layers [18,19,20,21,22].

The objective of this work is to present a method to reduce delamination between low-affinity polymer materials, which can be used to scale up bilayer membranes. Variables that govern delamination and the mechanism(s) that influence delamination between bilayer membrane layers are identified. Two low-affinity polymers, polysulfone (PSf) and polyvinylidene fluoride-co-hexafluoropropylene (PVDF-HFP), are chosen as the base membrane polymeric materials. Although PVDF-HFP is flexible, it has low rigidity, making it susceptible to degradation in alkaline conditions. Therefore, a bilayer membrane composed of both PVDF-HFP and PSf presents the opportunity to withstand both alkaline and acidic environments while also resisting oxidative conditions because the low rigidity of PVDF-HFP can be supported by PSf, if delamination between the two layers is successfully addressed. To enhance delamination resistance and adhesion, additive polymers such as poly(methyl methacrylate) (PMMA) and polydopamine (PDA) are incorporated into the membrane structure due to their higher affinity to PVDF-HFP and PSf. PMMA is utilized as a grafting material for these polymers, offering the potential to function as an interfacial compatibilizer. PDA possesses adhesive properties that could promote layer adhesion between PVDF-HFP and PSf. They are coupled with co-solvents, Polarclean (PC) and γ-Valerolactone (GVL), between low- and high-affinity polymers and between layers to determine how dope solution movement behaves at the interface with low- and high-affinity polymers and between layers, and how it affects the adhesion of the bilayer membranes. The affinity of the materials is predicted with the relative energy difference (RED) calculation, using the Hansen solubility parameter (HSP) of each polymer. The low affinity leads to a high possibility of delamination. This work enables scalable fabrication of bilayer membranes using low-affinity polymers via a scalable manufacturing process that couples slot-die coating on an R2R system followed by non-solvent-induced phase inversion (NIPS).

## 2. Materials and Methods

### 2.1. Polymers and Solvents

PSf and PVDF-HFP were supplied by Arkema (Radnor, PA, USA). PSf and PVDF-HFP serve as base polymers for the bilayer membranes due to their low affinity towards each other. In addition, PSf demonstrates excellent mechanical stability, resistance to alkaline environments, and thermal degradation. However, it is often susceptible to fouling due to its hydrophobic nature, and an uncontrolled phase inversion process may lead to the formation of macrovoids, which can compromise its structural integrity. Conversely, PVDF-HFP exhibits high resistance to acids and oxidative environments, along with greater compatibility with additive polymers. Dopamine hydrochloride and hydrogen peroxide were purchased from Sigma-Aldrich (St. Louis, MO, USA) and are precursors for PDA, an additive polymer. Poly(methyl methacrylate (PMMA), with a molecular weight (MW) of 450,000–550,000, was purchased from Alfa Aesar and served as another additive polymer. PC, supplied by Solvay (Houston, TX, USA), and GVL, purchased from Sigma-Aldrich, were used as solvents. To dissolve these polymers and formulate a castable dope solution, a solvent mixture of PC and GVL (Polarclean-GVL) in a 3:1 ratio was prepared [23,24,25].

### 2.2. Polymer Solution Formulation

For the PSf dope solution, PSf pellets were dissolved in a PC-GVL co-solvent at 80 °C and stirred magnetically for 24 h until a homogeneous solution was obtained. Similarly, PSf dope solution containing a PDA additive polymer was dissolved in a PC-GVL co-solvent with dopamine hydrochloride (DA) and hydrogen peroxide, then magnetically stirred for 24 h at 80 °C, using a one-pot method adapted from Mulyati et al. [26]. The dopamine hydrochloride in the mixture polymerizes to form polydopamine via oxygen generated by hydrogen peroxide once homogeneity is achieved. PVDF-HFP dope solutions are prepared by dissolving PVDF-HFP powder in PC-GVL at 80 °C under magnetic stirring for 24 h until a uniform solution is formed. When incorporating PMMA as an additive polymer, both PVDF-HFP powder and PMMA are dissolved in PC-GVL at 80 °C with magnetic stirring for 24 h. The concentration of PVDF-HFP is adjusted from 15% wt. to 8.4% wt. to ensure the viscosity of the dope solutions remains within a castable range, while keeping the total polymer content above 10% wt. for phase inversion. The maximum allowable loading of PMMA was 3.17% wt. to prevent the deformation of the underlying bottom layer from the top layer due to viscosity. This correlates to a viscosity below 3.7 Pa·s at a shear rate of 45 s^−1^. Additionally, PVDF-HFP dope solutions with PDA additive polymer are prepared by dissolving PVDF-HFP powder, using the same method employed in the PSf-PDA dope solution, DA, and hydrogen peroxide in Polarclean-GVL at 80 °C under magnetic stirring for 24 h. The concentrations of the dope solution compositions are shown in Table 1.

### 2.3. Bilayer Film Fabrication

For the fabrication of bilayer membranes, a dual-layer slot-die integrated within an R2R system was employed. The schematic of the slot-die with the R2R system is illustrated in Figure 1a. The dual-layer slot-die, depicted in Figure 1b, consists of two separate slots divided by a mid-die, enabling the simultaneous deposition of the dope solution. Initially, Slot 1 deposits the bottom layer, followed by Slot 2 depositing the top layer, allowing both solutions to contact each other in the meniscus. The dual-layer slot-die transfers the dope solutions within a sealed environment, thereby preventing exposure to ambient vapor prior to deposition. This closed-environment operation minimizes time-dependent variables, and precise regulation of the flow rate facilitates accurate control of the dope solution thickness, which is critical for the fabrication of thin membranes. The dope solutions are deposited on top of the moving glass substrates at a specific speed to form the bilayer structure. In this study, a PSf-based dope solution and a PSf-based dope solution with an additive polymer were deposited through Slot 1 for the bottom layer. For the top layer membrane, a PVDF-HFP-based dope solution with an additive polymer was deposited through Slot 2. The PVDF-HFP membranes are coated as the top layer because the PVDF-HFP polymer and its dope solution have lower surface tension than PSf. Having a lower surface tension dope solution on the top layer mitigates a dewetting defect, where the breaking of the film occurs due to surface tension flow [27]. In addition, PVDF-HFP is more hydrophobic compared to PSf, where having more hydrophilic membrane materials on the bottom layer can mitigate water coagulation at the interface, which could also lead to delamination of the bilayer membrane [28].

Following the simultaneous deposition of dope solutions under the parameters specified in Table 2, the resulting coated film is immersed in a deionized (DI) water bath to induce phase inversion, also known as non-solvent-induced phase separation (NIPS). During this process, pore formation occurs as solvent and non-solvent exchange due to differences in chemical potential, as shown in Figure 1c. The phase-inverted bilayer membrane samples are stored in DI water for a period of 24 h to eliminate any residual solvent prior to characterization, ensuring the removal of trapped solvents within the membrane matrix.

### 2.4. Morphological Characterization

Scanning electron microscopy (SEM) images were obtained using a Hitachi SU8230 (Hitachi High-Tech Corp., Hitachi-naka, Japan) and a Phenom XL microscope (Thermo Fisher Scientific, Eindhoven, The Netherlands), operated at voltages ranging from 5 kV to 10 kV, to examine the cross-section of the samples. The samples were cut with a razor blade due to delamination of the sample when dipped in liquid nitrogen to fracture. Gold or a gold-platinum alloy was sputtered onto the sample before imaging, using either a Quorum Q-150V ES Plus (Quorum Technologies, Laughton, UK) or a Cressington 108 Sputter Coater (Quorum Technologies, Laughton, UK), for increased visibility of low-conductivity polymer materials used in this study.

### 2.5. Adhesion Tests

The adhesion of the layers was assessed through the ASTM D3359-B standard test, where the degree of adhesion is classified based on the percentage of delaminated segments within a 10 mm by 10 mm grid [29]. In total, 25.4 mm Scotch 610 adhesive tape is applied over the area and then peeled from the surface to evaluate the delamination on a scale of 0B–5B, where 5B means no delamination is exhibited.

### 2.6. Filtration Experiments

Filtration tests were performed using dead-end filtration at a pressure of 60 psi. The performance criteria were the flux and rejection of the solute. The water flux was calculated as J = V/(A × t), where V is the volume of filtered water, A is the area of the membrane, and t is the total time of the filtration. The permeation was calculated with Lp = J/ΔP where ΔP is the pressure. The membranes were precut to the size of the filtration cell, with an area of 13.4 cm^2^, and solute–water solutions were added. The pressure was applied to the cell, and the solution was allowed to filter through the membrane. Conductivity tests were performed to measure the rejection rate of the solute. In total, 100 ppm sodium chloride (NaCl), calcium chloride (CaCl), and Bovine Serum Albumin (BSA) were used as the monovalent ion, divalent ion, and protein rejection medium, respectively. The rejection (R) was calculated as R = (1 − C_p_/C_f_) × 100%, where C_p_ and C_f_ are the concentrations of the permeate and feed solution, respectively. NaCl has a size of 3.6 Å, CaCl_2_ has a size of 4.1 Å, and BSA has a size of 71 Å.

### 2.7. FTIR Characterization

The presence of PDA in the samples with PDA as an additive was analyzed using Fourier transform infrared (FTIR) spectroscopy. Spectra were recorded on a Thermo Fisher Nicolet 6700 spectrometer (Thermo Fisher Scientific, Madison, WI, USA) equipped with an attenuated total reflectance (ATR) accessory containing a diamond crystal. Prior to the characterization, PDA bilayer samples were completely dried for 24 h. The sample was placed directly onto the ATR crystal and clamped down to ensure uniform optical contact for both the top and bottom layers. All spectra were collected in the mid-infrared region from 4000 to 400 cm^−1^ at a resolution of 4 cm^−1^, with an accumulation of 32 scans per measurement. Background spectra collected under ambient conditions without a sample were subtracted automatically by the OMNIC software (v8.1) settings.

## 3. Results and Discussion

### 3.1. Cahn–Hilliard Equation: Interface Diffusion

The absence of diffusion at the interface, which results in delamination when two immiscible polymers are in contact, can be elucidated through the derivation of the Cahn–Hilliard equation. This equation, formulated by Cahn and Hilliard, describes phase separation or spinodal decomposition [30]. As an energy-based model, it effectively simulates the behavior of two immiscible materials and their interactions in a fluid state when in contact.

(1) $$F=\int \left[f\left(\phi \right)+\frac{1}{2}\kappa {\left|\nabla \phi \right|}^{2}\right]dV$$

where *F* is free energy. The right-hand side of Equation (1) delineates the free energy characteristics for both immiscible and miscible systems, where $f\left(\phi \right)$ is the bulk energy and $\frac{1}{2}\kappa {\left|\nabla \phi \right|}^{2}$ is the interface energy between two bulk fluids, where ϕ is the concentration between two dope solutions and κ is the gradient energy coefficient. $f\left(\phi \right)$ is indicative of the bulk free energy, a crucial factor that influences separation behavior in these materials. For immiscible materials, the fluid behavior is typically captured by the double-well potential as illustrated in Figure 2a. In this framework, the curve features two distinct lowest-energy states located at concentrations of 0 and 1. These minima correspond to phases where the materials are completely separated, promoting spontaneous phase separation. It is important to note that while the concentrations might not strictly reach 0 and 1, they are often found in close proximity to these values. In contrast, the miscible case is represented by a single-well potential, depicted in Figure 2b. This scenario features a single minimum at a concentration of 0.5, indicating that the lowest energy state of the system is achieved when the two fluids are mixed in equal proportions. Consequently, this arrangement facilitates spontaneous mixing of the fluids, as they naturally gravitate toward this equilibrium state.

The interface energy $\frac{1}{2}\kappa {\left|\nabla \phi \right|}^{2}$ increases in energy when the interface gets sharper. Consequently, a balance exists between the tendency of immiscible fluids to separate and the interface energy, which prevents the interface thickness from becoming infinitesimally small. This relationship is exemplified by the chemical potential equation given in Equation (2), which is a derivative of the free energy function. In the case of miscible fluids, the spontaneous mixing driven by the single-well potential results in low free energy associated with diffusion-induced concentration gradients. Such low free energy reduces the interface energy to near zero, leaving primarily the $\frac{df}{d\phi }$ associated with diffusion.

(2) $$\frac{\delta F}{\delta \phi }=\mu =\frac{df}{d\phi }-\kappa \nabla^{2}\phi$$

where μ is the chemical potential.

Equation (3) is the Cahn–Hilliard Equation, which describes how the change in free energy and the mobility term affect the concentration over time.

(3) $$\frac{d\phi }{dt}=\nabla \cdot \left[M\nabla \left(\frac{df}{d\phi }-\kappa \nabla^{2}\phi \right)\right]$$

where M is the mobility term. When *M* is multiplied by the change in chemical potential, $\nabla \left(\frac{df}{d\phi }-\kappa \nabla^{2}\phi \right)$, the flux of the system is given. In the case of immiscible fluids, spontaneous separation occurs until the interfacial energy balances the separation between layers. Therefore, the flux exists, but the directionality of the flux is in the direction of separation of the solutions rather than the direction of mixing. Consequently, in an immiscible fluid system, there is minimal mixing between the fluids, resulting in a distinct interface, even if they share the same solvent. This model suggests why delamination of the membrane between the low-affinity dope solution and the fluid behavior of the immiscible dope solution exists; however, it does not accurately predict the interface thickness or how exactly the fluid will behave, since the bulk energy profile cannot be accurately modeled.

#### Relative Energy Difference (RED) Value: Dope Solution Affinity

The term $f\left(\phi \right)$ relies on the miscibility of the materials involved, which can be predicted using the RED value, Equation (4), using Ra, the Hansen solubility parameter (HSP) distance, and *Ro*, the interaction radius was calculated with HSP values, as seen in Table 3. The $\delta_{ds}$ and $\delta_{dp}$ represent the dispersion bond energies of the polymer and solvent, respectively, while $\delta_{ps}$ and $\delta_{pp}$ denote the polar bond energies of the polymer and solvent, respectively. Lastly, $\delta_{hs}$ and $\delta_{hp}$ are the hydrogen bond energies of the polymer and solvent, respectively. RED values over 1 indicate low affinity between polymers; that is, their mixing likely exhibits double-well behavior and phase separation instead of spontaneous mixing. However, the casting process of the membrane requires a dope solution to be cast on top of the substrate, which requires the polymer to be dissolved in a solvent. Dissolving the polymer in the solvent will change the HSP due to mixing with the solvent. HSP values between dope solution mixtures become similar with the addition of the same solvent, which can be elucidated with Equations (6)–(8). HSP values of the dope solution are calculated using Equations (6)–(8), where $\Phi_{s}$ and $\Phi_{p}$ are the volume fractions of the polymer and solvent, respectively, while $\delta_{dmix_{1}}$, $\delta_{dmix_{1}}$, and $\delta_{dmix_{1}}$ represent the dispersion bond energy, polar bond energy, and hydrogen bond energy of the dope solution, respectively. However, it does not necessarily ensure spontaneous mixing between dope solutions due to low affinity between polymer molecules that are dissolved in the dope solution [31]. Low-affinity polymer molecules tend to repel each other, leading to phase separation or precipitation instead of mixing. This phenomenon occurs even when the HSP values of the dope solutions are relatively close. For instance, the dope solutions of PSf-PC-GVL and PVDF-HFP-PC-GVL exhibit HSP values of 17.13, 11.52, and 8.67, respectively, and 16.3, 12.13, and 9.15, respectively, as detailed in Table 3.

To investigate the behavior of two doping solution systems in relation to their bulk free energy profiles, a heuristic approach was employed, involving the mixing of two distinct doping solutions. This method allows for an assessment of whether the combined system exhibits characteristics of a double-well or single-well free energy. Observing whether the mixture separates into phases or mixes homogeneously provides insight into the bulk free energy. Phase separation upon mixing indicates that the bulk free energy is similar to the double-well equation, which minimizes diffusion between the dope solutions. Conversely, a homogeneous mixture suggests that the bulk free energy aligns more closely with the single-well equation, maximizing diffusion between the solutions.

(4) $$RED=\frac{Ra}{R_{0}}$$

(5) $$Ra=\sqrt{4{\left(\delta_{dp}-\delta_{ds}\right)}^{2}+{\left(\delta_{pp}-\delta_{ps}\right)}^{2}+{\left(\delta_{hp}-\delta_{hs}\right)}^{2}}$$

(6) $${{\delta_{dmix_{1}}=\Phi }_{s_{1}}\delta }_{ds_{1}}+{\Phi_{p_{1}}\delta }_{dp_{1}},$$

(7) $${{\delta_{pmix_{1}}=\Phi }_{s_{1}}\delta }_{ps_{1}}+{\Phi_{p_{1}}\delta }_{pp_{1}},$$

(8) $${{\delta_{hmix_{1}}=\Phi }_{s_{1}}\delta }_{hs_{1}}+{\Phi_{p_{1}}\delta }_{hp_{1}}$$

### 3.2. Low Affinity Layer Delamination and Relative Energy Difference

The layer delamination observed, as depicted in Figure 3a for Sample 1 (see Table 2), can be attributed to several factors commonly associated with coating processes, including low interfacial affinity between the materials, differences in their shrinkage ratios, and mismatches in hydrophilicity. While the first two factors contribute to delamination, the predominant cause in this context is the limited adhesion stemming from the inherently low affinity between the bilayer membrane materials used. The adhesion mechanism involves various intermolecular forces such as hydrogen bonding, molecular entanglement, and crosslinking. However, since crosslinking was not implemented in the materials used in this study, the primary adhesion forces are hydrogen bonds and entanglement of polymer chains. Hydrogen bonding occurs between high-polarity molecules that attract each other despite not being miscible. Molecular entanglement arises when polymers are miscible, leading to intertwining of their chains and resulting in adhesion [14].

The Cahn–Hilliard equation implies that low-affinity materials do not bond effectively due to their separation caused by a lack of diffusion or penetration. The NIPS process exemplifies how two-phase separation induces nucleation of the polymer-lean phase upon the introduction of a non-solvent. Likewise, the separation of materials prevents molecular entanglement by limiting mixing and diffusion between low-affinity polymers.

The PSf and PVDF-HFP dope solutions presented a cloudy mixture upon combining, which suggests that the two solutions are not fully mixing but are rather undergoing phase separation. This observation indicates that the PSf and PVDF-HFP system possesses a bulk free energy profile consistent with a double-well potential, as illustrated in Figure 4a. Thus, the spontaneous separation hampers the diffusion of polymer molecules, which helps to explain the occurrence of delamination between the PSf and PVDF-HFP bilayer membrane.

As discussed in Section 3, diffusion adhesion between low-affinity polymers is limited, leading to delamination. In addition, sharing the same solvent between dope solutions does not ensure mixing between dope solutions despite having similar HSP values (see Table 3). Consequently, modifying the solvent or polymer volume cannot guarantee the miscibility of the dope solution for increasing adhesion by increasing affinity. In this investigation, the additive polymer approach was selected because additive polymers have the potential to bridge the RED gap between PVDF-HFP and PSf, since the addition of an additive polymer can alter the HSP at the interface. Furthermore, an appropriately chosen additive polymer can facilitate hydrogen bonding between the PSf and PVDF-HFP membrane layers, thereby enhancing adhesion. PMMA and PDA were selected as additive polymers due to their favorable properties related to layer adhesion.

### 3.3. Layer Adhesion with Additive Polymer

#### 3.3.1. PMMA Additive Polymer

PMMA has the capability to be mixed with PSf, with a low RED value (see Table 3) between PSf and PMMA, which indirectly indicates that PMMA and PSf have a higher affinity for each other than that of PVDF-HFP and PSf (see Table 3) [35]. The poor mixing between PVDF-HFP and PSf is further validated by a mixture of PSf and PVDF-HFP dope solution showing immiscibility when mixed, as shown in Figure S1. PMMA would not necessarily induce spontaneous mixing but could act as an interface tension mitigator that reduces interfacial tension between PVDF-HFP and PSf, since the higher affinity of PMMA drives monomers into the PSf from PVDF-HFP. PVDF-HFP exhibits a low affinity for PSf, resulting in repulsive interactions. This lack of affinity hinders effective mixing. Conversely, PMMA, which is co-dissolved with PVDF-HFP, possesses a comparatively higher affinity for PSf. As a result, PMMA migrates closer to PSf, promoting better interfacial interaction and lowering interfacial energy. This results in PMMA acting similarly to a surfactant, leading to a broader interface thickness due to lowered free energy according to the Cahn–Hilliard equation, which facilitates partial penetration or blending of the PMMA into the PSf, resulting in better adhesion [36,37,38]. In addition, PMMA has the capability to induce hydrogen bonding and is miscible with PVDF-HFP, which has the potential to facilitate adhesion between layers. PMMA was added to the PVDF-HFP dope solution as a layer-enhancement additive polymer. Sample 2 bilayer with PMMA employed as an additive polymer for the top layer PVDF-HFP is shown in Figure 3b. Unlike the bilayer sample without any additive polymer, bilayer Sample 2 with PMMA as the additive polymer does not show noticeable delamination in the top-view camera image. The PMMA, which was incorporated into the PVDF-HFP molecule, could have effectively altered the RED value of the dope solution at the interface. A lower RED value at the interface indicates higher affinity locally, potentially shifting the bulk free energy, $f\left(\phi \right)$, from a double well closer to a single well, thereby facilitating interpenetration of PMMA between dope solutions. The penetration of PMMA monomer due to increased affinity can promote entanglement between polymer molecules at the interface, enabling them to anchor together and enhance adhesion between the two layers, resulting in a broader interface [39]. Enhanced layer adhesion of the bilayer membrane with PMMA as an additive polymer is believed to be due to enhanced entanglement at the interface, as illustrated in Figure 3b as a picture of the top view and Figure 4c as an SEM cross-section of the thickness. The top-view image indicates that, relative to the pristine PVDF-HFP and PSf bilayer membranes, as shown in Figure 3a, wrinkling is significantly reduced or eliminated. This reduction in wrinkling is attributed to the high glass transition temperature, 105 C, of the incorporated PMMA, which was added to the PVDF-HFP dope solution. High glass transition temperatures often make polymers exhibit more rigid characteristics [40]. Overall, the elevated glass transition temperature of PMMA enhances the rigidity of PVDF-HFP, which exhibits a glass transition temperature of −40 °C, when incorporated into the PVDF-HFP membrane [41]. The SEM cross-sectional image, Figure 4c, illustrates that there is no delamination between the top and bottom layers, as evidenced by the absence of observable voids or delamination at the interface, unlike what is shown in Figure 4b. The thickness of the membrane sample with PMMA was 81.1 µm with a standard deviation of 6.61 µm for the top layer, PVDF-HFP-PMMA, and was 141.7 µm with a standard deviation of 4.6 µm for the bottom layer, PSf. The incorporation of PMMA facilitated the ability of the layers to withstand shrinking stresses, where the top layer and bottom layer have different shrinking speeds and ratios, during phase inversion—analogous to drying stress in film coating. Higher affinity at the interface results in a broader interface between the two dope solutions. Additionally, a broader interface resulted in a less pronounced interface, as shown in Figure 4c, compared to the pristine bilayer sample, Sample 1, shown in Figure 4b, without any additive polymer. A broader interface could be validated by the gradual decrease of fluorine content from the top layer to the bottom layer and the increase in sulfur content from the top layer to the bottom layer. A steeper decrease and increase in the fluorine and sulfur content were observed in the bilayer sample without PMMA. The PMMA could have aided the penetration of the PVDF-HFP into the PSf layer, resulting in gradual changes in the composition of the fluorine and sulfur content. However, there are limitations with EDS measurements, whereas XPS measurements at the interface could validate the penetration in future studies by detecting the PMMA directly.

##### PMMA Additive Polymer Pore Morphology

There is a pronounced variation in pore architecture, where the PVDF-HFP combined with the PMMA layer—containing the added polymer—demonstrates markedly larger pore structures compared to the unmodified PVDF-HFP layer qualitatively, as shown in Figure 4b,c. Although PMMA generally does not act as a pore-forming agent like polyvinylpyrrolidone (PVP) due to its hydrophobic nature, its inclusion in PVDF-HFP serves as a pore former because PVDF-HFP is more hydrophobic due to its fluorine content. As a result, the PVDF-HFP-PMMA dope solution is less hydrophobic compared to the pristine PVDF-HFP dope solution. This correlates with the dope solution being more hydrophilic with the added PMMA additive polymer. Consequently, the phase separation process is altered, resulting in different pore density and morphology. It is believed that PMMA accelerates phase separation, promoting the formation of more macropores. Similarly, the PSf layer exhibits morphological modifications when contrasted with pristine PSf samples. A bilayer sample incorporating PMMA as an additive polymer shows increased macropores in the PSf layer at the base compared to a bilayer without additives, as illustrated in Figure 4c and Figure 4b, respectively. This phenomenon could be attributed to the rapid phase separation induced by the PVDF-HFP layer with PMMA, which enhances the phase inversion rate of the underlying PSf layer. This expedited phase inversion, facilitated by accelerated solvent–nonsolvent exchange, leads to the formation of additional macropores. These findings suggest that alterations in the phase-inversion conditions of the top membrane layer can influence the pore morphology of the underlying layer, as solvent and non-solvent exchange dynamics are directly affected by the initial phase inversion of the top layer.

A significant amount of macropores has the potential to weaken the polymer matrix of the membrane. A weaker matrix can be problematic during membrane operation, especially due to the pressures involved in the operation. Therefore, an appropriate amount of additive polymer or an additive polymer type needs to be considered for the use of layer adhesion.

#### 3.3.2. Polydopamine Additive Polymer

Another additive polymer was explored, polydopamine (PDA). PDA was added to enhance the layer adhesion. PDA is a nature-inspired material that has adhesive characteristics due to its molecular structure. PDA molecules have multiple hydroxyl branches that can hydrogen bond with other materials; they can also form π-π stacking and have the ability to form adhesion with van der Waals forces [42]. These properties can aid adhesion between two polymers that do not mix or adhere; however, PDA may not change the affinity between the polymers when added to the dope solution in its fluid state if a small amount is used. According to Equation (6), the HSP of the mixture depends on the ratio of the material amount in the mixture, which indicates that a very low amount of additive polymer will have minimal effect on the HSP of the mixture. Here, 1.5% wt. of PDA precursors were used; therefore, a clean interface is expected, which indicates low diffusion at the interface. However, PDA molecules that are in the dope solution help with adhesion during the phase inversion, when the polymer dope solution phase changes into a solid. PDA molecules, embedded in the polymer matrix and not fully washed out by DI water, can form hydrogen bonds with PSf and PVDF-HFP at the interface, also known as chemical bonding adhesion [39]. In addition, PDA is a hydrophilic polymer that can improve membrane permeability and reduce fouling, unlike PMMA.

A top camera view of bilayer Sample 3, which incorporates PDA, is depicted in Figure 3c. Sample 3 has a brown tint and consists of more wrinkles compared to bilayer membrane samples that have PMMA as an additive polymer, i.e., Sample 2. The PDA has a lower glass transition temperature than PMMA; therefore, when PDA is added as an additive polymer, the fabricated membrane is more supple and more prone to wrinkling because it cannot withstand the shrinking forces that form during phase inversion, similar to shrinking as many wet films dry via convection, unlike higher glass transition temperature PMMA. Furthermore, every material has a different phase inversion speed due to its thermodynamic characteristics, where phase inversion speed disparity emerges [43]. The structural formation of the bilayer itself can create a phase inversion speed difference, where the top layer has initial contact with the non-solvent phase, thereby inverting faster than the bottom layer because of its proximity to the driver (DI water) during the solvent and non-solvent exchange. Given this difference in the rate of shrinkage during phase inversion, it exacerbates wrinkling because one layer will finish phase-inverting and turn into a solid state, while other layers continue shrinking during the ongoing phase inversion process. Consequently, wrinkles will be created because there is a shrinkage limit that will be met in the top layer as the membrane fully phase-inverts. Even with wrinkling and shrinkage, both layers were able to adhere with embedded PDA molecules, possibly with hydrogen bonding at the interface area, unlike the bilayer sample without an additive polymer, since the amount of PDA added does not increase the affinity for diffusion adhesion.

The SEM cross-sectional image, shown in Figure 5a, further demonstrates enhanced adhesion between the layers at their interface. Consistent with observations in PMMA samples, no voids are evident at the interface, indicating strong adhesion. In contrast, PDA exhibits a distinct phase inversion effect, with samples displaying a more densely packed pore structure compared to the PMMA sample, Sample 2, qualitatively, as shown in the SEM images in Figure 4c and Figure 5a. This increased pore density also accounts for the greater shrinkage observed in PDA bilayer samples, as larger pores reduce the overall density of the polymer, potentially decreasing shrinkage, whereas tightly packed pores increase density and may lead to higher membrane shrinkage. PDA bilayer samples exhibited a thickness of 50.5 µm with a standard deviation of 4.6 µm for the top layer, PVDF-HFP-PDA, and 127.5 µm with a standard deviation of 3.3 µm for the bottom layer, PSF-PDA.

The PDA bilayer sample, Sample 3, exhibits a distinct interfacial morphology compared to the PMMA bilayer sample, with a more clearly defined interface, as observed in Figure 5a. This difference arises from a different layer adhesion mechanism associated with PDA, which enhances hydrogen bonding between layers but does not significantly alter HSP to promote mixing, consistent with $f\left(\phi \right)$ being a double-well equation where spontaneous phase separation persists. The low amount of PDA addition has minimal impact on the HSP of the PVDF-HFP-PDA solution due to the low ratio of PDA. Therefore, the low affinity between PVDF-HFP-PDA and PSF-PDA persists, which correlates with the two dope solutions having double-well bulk free energy. Despite the low degree of mixing and a well-defined interface, the bilayer sample did not show signs of layer delamination, as depicted in the top view (Figure 3c) and in the cross-sectional SEM image (Figure 5a).

##### Polydopamine Additive Polymer Pore Morphology

The PDA has a pronounced influence on pore density, resulting in a greater number of larger pores in the PVDF-HFP + PDA layer, as shown in Figure 5a, relative to pristine bilayer samples, as shown in Figure 4b, lacking an additive polymer such as PMMA. Unlike PMMA, PDA functions as a pore former due to its water miscibility, acting as a non-solvent. Similar to PVP, PDA leaches out during phase separation, facilitating the formation of larger pores. The leaching of the PDA was evident during the NIPS process, where the non-solvent color changed to slightly brown over time when the membranes were immersed. Therefore, FTIR measurements were taken on the top and bottom layers of the PDA-added bilayer membrane to validate that PDA had not fully leached out during the phase separation process, as shown in Figure 5b,c. The FTIR measurements for the top layer, PVDF-HFP-PDA, detected a signature of hydrogen bonding at 3283 cm^−1^, which indicated the presence of polydopamine. In the FTIR measurements of the bottom layer, the PSf-DA layer, a similar small peak was detected around 3293 cm^−1^, but the signature was much weaker and broader than that of PVDF-HFP-PDA. A weaker signature of PDA in the PSf-PDA layer could be due to intermolecular hydrogen bonding with sulfonyl and ether linkages in PSf molecules. Furthermore, the hydrophilic character of PDA renders the membrane more hydrophilic, accelerating phase separation and increasing pore size [44]. At the interface, the pore structure appears homogeneous, indicating a slower phase inversion or demixing process from the interface to the underlying layers, as shown in the SEM image in Figure 5. The presence of the top layer additionally impedes solvent and non-solvent exchange in the lower layers. The slower phase inversion, driven by reduced solvent and non-solvent exchange rates, likely accounts for the homogeneous polymer structure observed at the interface.

### 3.4. Adhesion

The effectiveness of the layer adhesion using an additive polymer that changes the RED value to have a higher affinity or induce hydrogen bonding between layers was assessed using the ASTM D3359-B adhesion test. The pristine sample, shown in Figure 6a, exhibited complete delamination of the PVDF-HFP layer when the layer adhesion test was conducted. Low affinity between the polymers created such delamination due to the lack of intermixing between the polymers. For the PMMA sample, layer adhesion was assessed using two samples to measure the adhesion properties. The average delamination area was found to be 18%, with a standard deviation of 5%, resulting in adhesion of 2B. The PMMA, added to the PVDF-HFP layer to increase the affinity between polymers, improved the adhesion from 0B to 2B classification by diffusion adhesion at the interface at the microscopic level, as shown in Figure 6b. The more rigid characteristics of the PMMA enabled PVDF-HFP to delaminate cleanly, without tearing or partial delamination. For the PDA sample, layer adhesion was evaluated using three samples. The average delamination area recorded was 19%, with a standard deviation of 4%. This also indicates a 2B adhesion level, resulting in delamination effects ranging from 15% to 35%, shown in Figure 6c. In addition, a more wrinkled structure that forms in the PDA sample could result in elevated adhesion because the wrinkles create more surface area at the interface for polymer adhesion, leading to higher adhesion than a flat interface.

### 3.5. Filtration Data

The adhesion results indicate that the bilayer sample incorporating PMMA and PDA demonstrated comparable adhesion values, despite differing adhesion mechanisms. However, for actual membrane performance, there is a clear advantage for PDA over PMMA. PMMA, being a hydrophobic polymer, results in a hydrophobic membrane that generally exhibits lower permeability and is more susceptible to fouling. Since the PVDF-HFP dope solution added with PMMA has PMMA monomers distributed throughout the membrane, hydrophobicity persists. Furthermore, the cross-sectional image of Sample 2, as shown in Figure 4c, reveals that the PVDF-HFP-PMMA layer, which serves as the top layer, contains a large macropore due to PMMA, posing a risk of collapse during filtration. Conversely, PDA is a hydrophilic material, and membranes fabricated using PDA have greater potential to remain hydrophilic, which helps alleviate issues related to low permeability and fouling. Consequently, bilayer samples featuring PDA are employed for membrane performance characterization, which includes assessments of permeability, BSA rejection, and divalent ion rejection.

Filtration data were collected for varying top layer flow rates during fabrication. The bilayer samples, with PDA used as an additive polymer, were designed with a thinner top layer to optimize flux for enhanced performance. As shown in Figure 7a, thicker top layers typically demonstrate lower permeation due to increased resistance. The resulting membranes exhibited ultrafiltration-range characteristics, achieving divalent ion rejection rates between 63.1% and 68.4%, as shown in Figure 7b, and BSA rejection rates between 94.8% and 97.5%, as shown in Figure 7c. Additionally, while thicker top layers yielded higher rejection rates, they also resulted in decreased permeability, consistent with Fick’s law. Higher rejection of the top-layer membrane, which is thicker, could also be attributed to the difference in phase inversion conditions due to the thickness of the top layer. The bilayer film structure of the cast dope solution before phase inversion allows most of the solvent and non-solvent exchange to happen at the free surface of the top layer. This leads to bottom layer solvent and non-solvent exchange happening through the top layer of the membrane, which, for the PDA sample, causes the bottom layer to phase separate more slowly, leading to a thicker interface. As a result, the top layer will determine the phase inversion condition for the bottom layer. This phenomenon is validated by the bilayer membrane with PMMA, Sample 2, which has a more porous top layer, PVDF-HFP + PMMA, thereby inducing more macropores in the bottom layer, PSf layer. Therefore, a thicker top layer could potentially reduce the phase inversion speed or solvent and non-solvent exchange, resulting in a thicker skin layer for the bottom layer [43,45,46]. A thicker skin layer at the bottom layer can also explain the lower permeability of the membrane and higher rejection with a thicker top layer.

## 4. Conclusions

Delamination resulting from the use of low-affinity materials in bilayer coatings has been explored for a variety of bilayer membranes fabricated using dual-layer slot-die coating on an R2R. The Cahn–Hilliard derivation expresses the behavior of the low-affinity dope solutions when they are in contact. Due to the double-well bulk free energy, $f\left(\phi \right)$, the dope solutions exhibit separation rather than mixing, which significantly reduces diffusion adhesion, resulting in delamination of the bilayer membrane. In addressing delamination due to low affinity, two methods of layer adhesion were employed using PSf and PVDF-HFP as base polymers for the membrane: diffusion mixing and chemical bonding facilitated by the addition of an additive polymer. In this study, the additive polymers PMMA and PDA were used to enhance the layer adhesion of the bilayer membrane by promoting both diffusion and chemical adhesion. The inclusion of the additive polymer in the PVDF-HFP dope solution improved diffusion mixing by manipulating HSP with PMMA, which changes bulk free energy behavior from double-well to single-well by increasing the affinity at the interface by acting as a surfactant or interface tension mitigator. The inclusion of PDA in both PSf and PVDF-HFP dope solutions enhanced chemical adhesion by increasing hydrogen bonding with PDA. Both approaches achieved layer adhesion of 2B, as characterized by ASTM D3359-B. It is important to highlight that the PMMA and PDA additive polymers influenced the pore morphology of the membrane due to their unique properties. Additionally, the thickness of the top layer of the membrane affects the morphology of the bottom layer because of changes in phase inversion conditions. This underscores the necessity for a strategic selection of both additive polymers and casting parameters based on the intended application of the membrane.

## Figures and Tables

**Figure 1:**
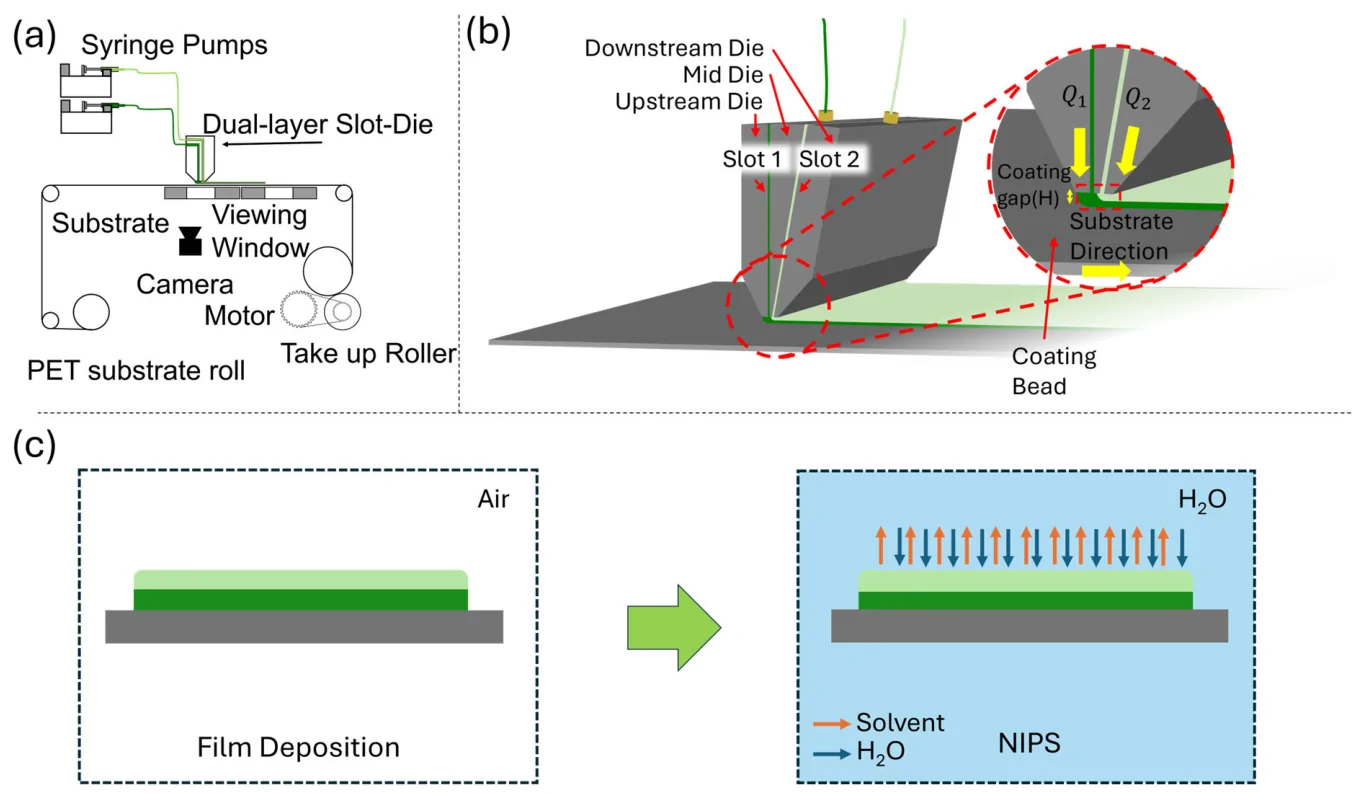
(**a**) Schematic of the R2R system with a dual-layer slot-die, (**b**) detailed schematic of the dual-layer slot-die, and (**c**) NIPS process for the pore-forming process; the arrows represent the direction of the solvent (orange) and non-solvent (blue) as solvent and non-solvent exchange during the NIPS process.

**Figure 2:**
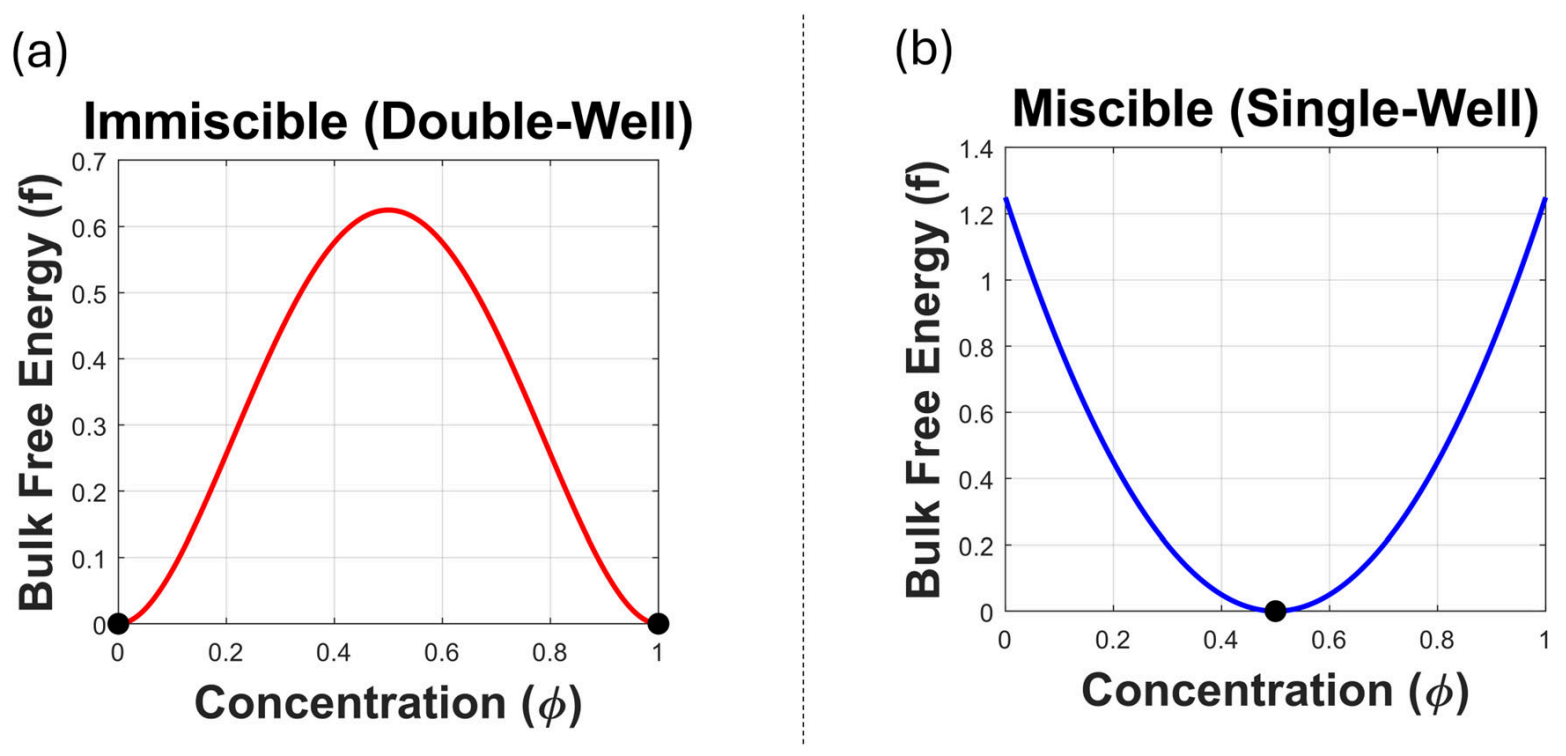
(**a**) Double-well equation for $f\left(\phi \right)$ term, and (**b**) single-well equation for $f\left(\phi \right)$ term.

**Figure 3:**
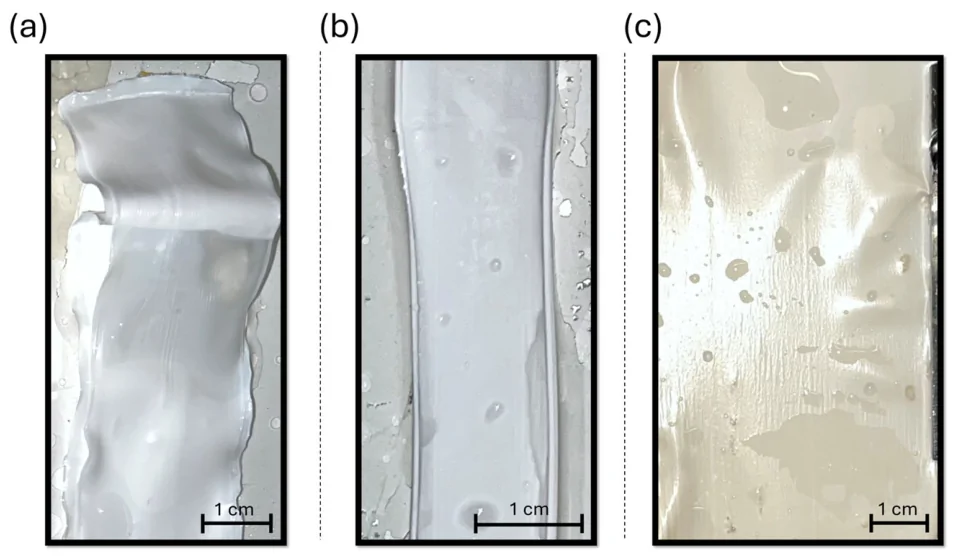
Top view camera image of the bilayer membrane samples: (**a**) PVDF-HFP (top layer), PSf (bottom layer); (**b**) PVDF-HFP + PMMA (top layer), PSf (bottom layer); and (**c**) PVDF-HFP + PDA (top layer), PSf + PDA (bottom layer).

**Figure 4:**
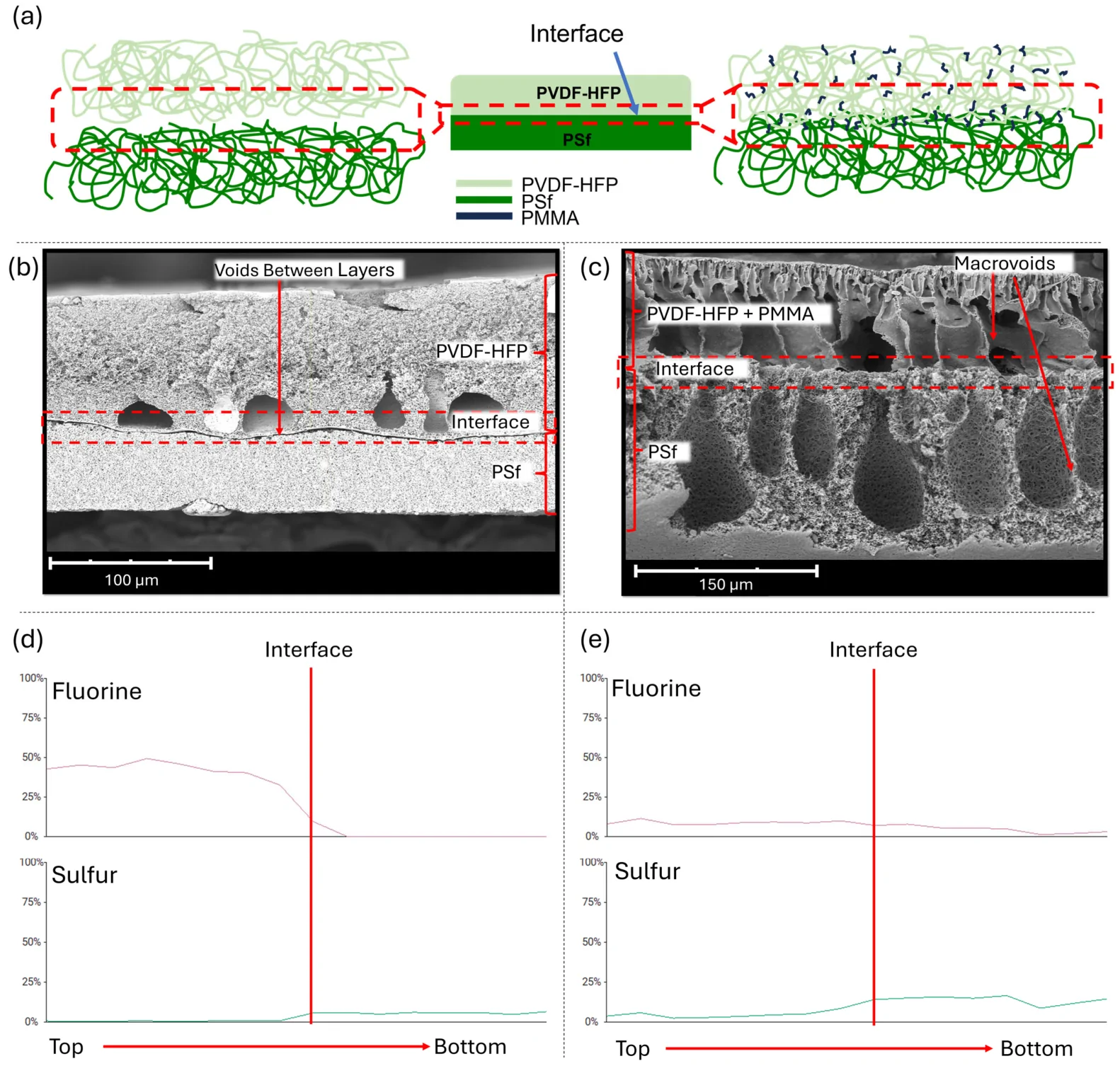
(**a**) Layer adhesion mechanism for PMMA, (**b**) bilayer Sample 1 without additive polymer, and (**c**) bilayer Sample 2 with PMMA as additive polymer. The EDS measurements for fluorine and sulfur content from top to bottom show the distribution in the top and bottom layers: (**d**) sample without PMMA and (**e**) with PMMA.

**Figure 5:**
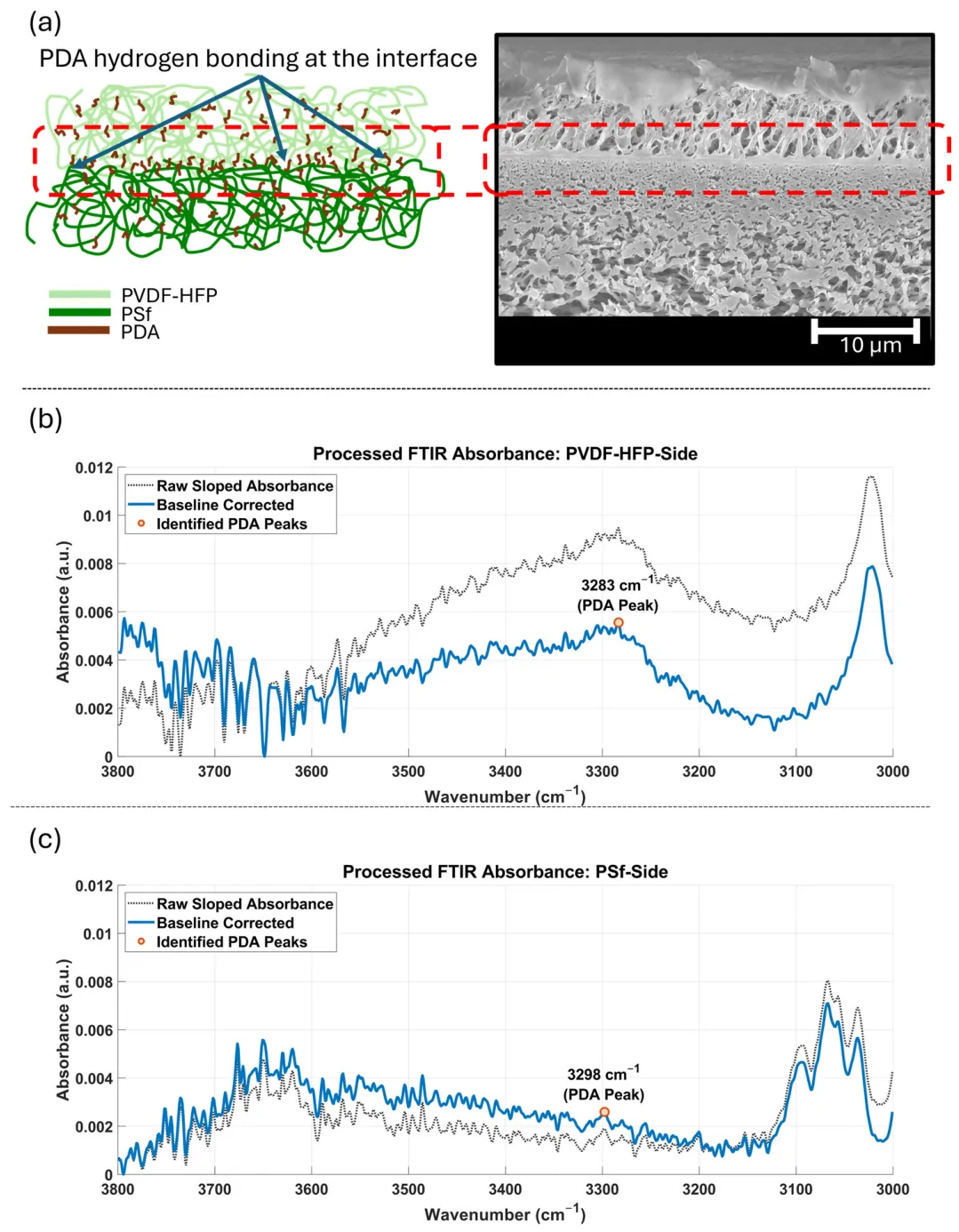
(**a**) PDA adhesion mechanism, SEM cross-section of the bilayer Sample 3 with PDA additive polymer. FTIR measurement of the bilayer membrane with PDA additive polymer for adhesion. (**b**) shows FTIR data for the PVDF-HFP-PDA layer, top layer, and (**c**) shows FTIR data for the PSf side, bottom layer.

**Figure 6:**
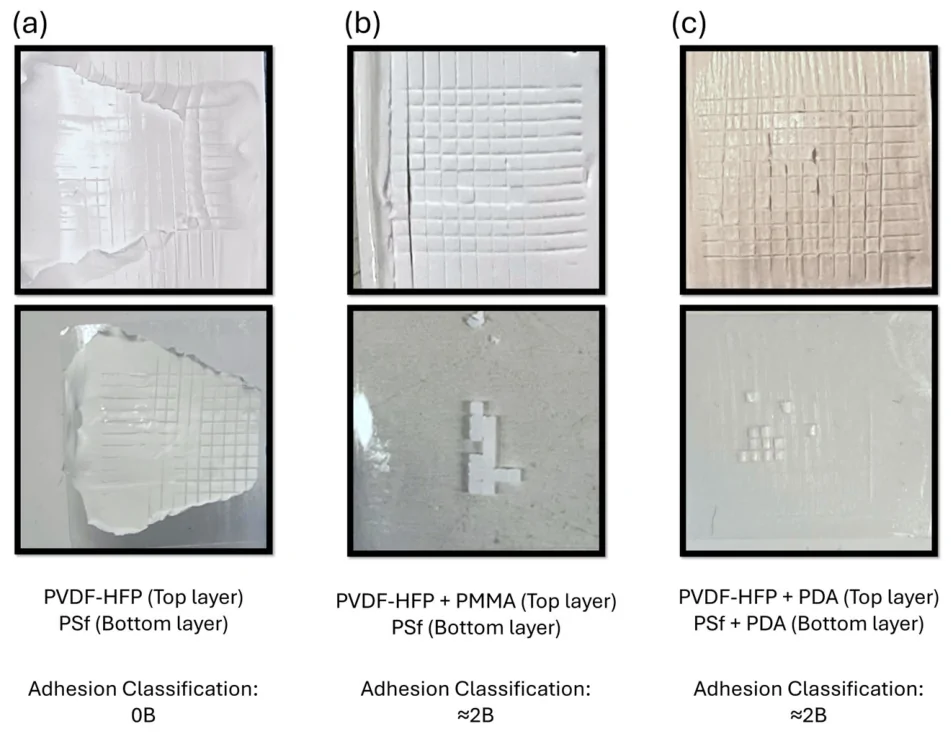
Top-view camera image of layer adhesion result with ASTM D3359-B test. The top images show a top-down camera view of the delaminated bilayer membrane, and the bottom images show the delamination of the top layer from the membrane onto the tape. (**a**) Sample 1, (**b**) sample 2, (**c**) sample 3.

**Figure 7:**
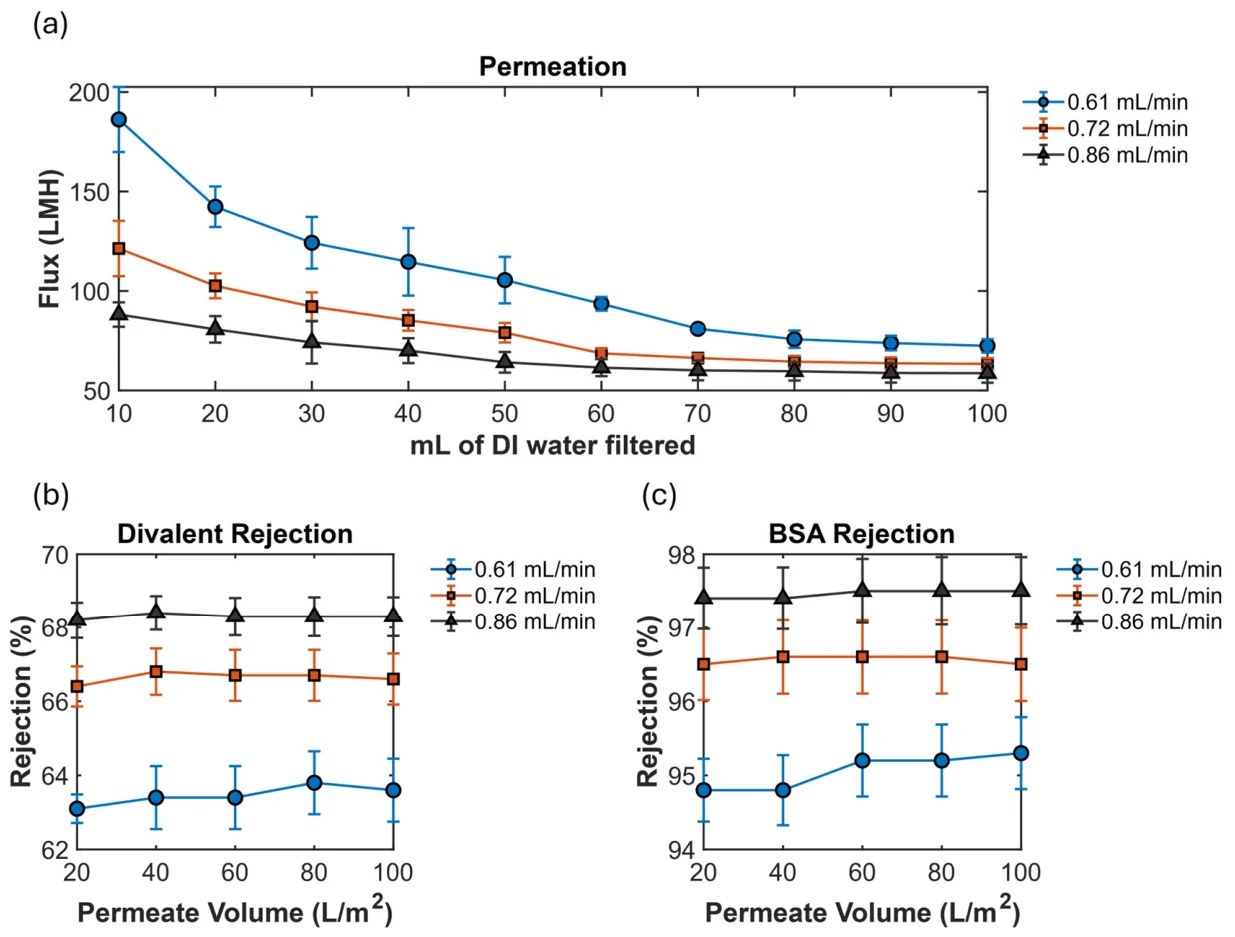
(**a**) Permeation data (each trial is 10 mL of DI water passing through the membrane), (**b**) divalent rejection data, and (**c**) BSA rejection data for varying top layer flow rate and membrane for varying top layer thickness, which is indicated with mL/min in the legend; the higher the flow rate, the thicker the top layer.

**Table 1:** Dope solution composition.

Dope Solution	PSf wt.%	PVDF-HFP wt.%	DA + H_2_O_2_ wt.%	PMMA wt.%	PC-GVL wt.%
PSf	17	0	0	0	83
PVDF-HFP	0	17	0	0	83
PSf-PDA	15.5	0	1 + 0.5	0	83
PVDF-HFP-PDA	0	15.5	1 + 0.5	0	83
PVDF-HFP-PMMA	0	8.4	0	3.17	88.43

**Table 2:** Dual-layer slot-die casting parameters for each sample, coating gap, and substrate speed are held constant at 300 µm (H) and 5.4 mm/s, respectively.

Sample	Top Layer	Bottom Layer	Flow Rate (Top) (Q_2_) (mL/min)	Flow Rate (Bottom) (Q_1_) (mL/min)	Slot-Die Width (in)
Sample 1	PVDF-HFP	PSf	2.4	2.4	1
Sample 2	PVDF-HFP-PMMA	PSf	2.4	2.4	1
Sample 3	PVDF-HFP-PDA	PSf-PDA	6.15	6.15	3

**Table 3:** HSP values for the polymers and solvents used for dope solutions for RED calculation between polymer–polymer, and HSP for dope solutions.

**Polymer**	$\delta_{dp}\;(MPa^{0.5})$	$\delta_{pp}\;(MPa^{0.5})$	$\delta_{hp}\;(MPa^{0.5})$	$R_{0}\;(MPa^{0.5})$	$R_{a}\;(MPa^{0.5})$	**RED**
(a) PSf [32]	19.7	8.3	8.3	8	(b) 11.25 (c) 0.4	(b) 1.41 (c) 0.56
(b) PVDF-HFP [33]	14.6	11.9	11.4	8.1	(a) 11.25 (c) 9.01	(a) 1.41 (c) 1.28
(c) PMMA [34]	18.6	10.5	5.1	8	-	-
**Solvent**	$\delta_{ds}$	$\delta_{ps}$	$\delta_{hs}$	$R_{0}$	$R_{a}$	
PolarClean [32]	15.8	10.7	9.2	-	-	-
GVL [32]	19	16.6	7.4	-	-	-
(d) PC-GVL (3:1)	16.6	12.2	8.8	-	-	-
**Dope Solution**	$\delta_{dmix_{1}}$	$\delta_{pmix_{1}}$	$\delta_{hmix_{1}}$			
(a) + (d) (17% wt.)	17.13	11.52	8.67	-	-	-
(b) + (d) (15% wt.)	16.3	12.13	9.15	-	-	-

## Data Availability

The data presented in this study are available on request from the corresponding author.

## Supplementary Materials

The following supporting information can be downloaded at https://www.mdpi.com/article/10.3390/membranes16100314/s1, Figure S1: The mixture of the PSf and PVDF-HFP dope solutions resulted in a cloudy appearance, indicating phase separation and immiscibility due to low affinity.
