# Supplementary material for: Mitigation of Delamination Between Bilayer Membranes with Low Affinity to Enable Roll-to-Roll Fabrication
Source: Membranes (Basel). 2026 Sep 25;16(10):314. doi: 10.3390/membranes16100314 (PMC13706712; doi:10.3390/membranes16100314)
Supplement: Supplementary file 1 [file membranes-16-00314-s001.zip › membranes-4507613-supplementary.pdf]

Supplementary information

Mitigation of Delamination between Bilayer Membranes with Low Affinity to Enable Roll-to-Roll Fabrication

Minwoo Jung[1], Eesh Kulshrestha[2], Isabel C. Escobar[2], Tequilla A. L. Harris[1*]

[1] *George W. Woodruff School of Mechanical Engineering, Georgia Institute of Technology, Atlanta, GA, 30332, USA*

[2] *Department of Chemical and Materials Engineering, Stanley and Karen Pigman College of Engineering, University of Kentucky, Lexington, Kentucky 40506, United States*

[*] **Corresponding authors:** Tequila Harris (tharris3@gatech.edu)

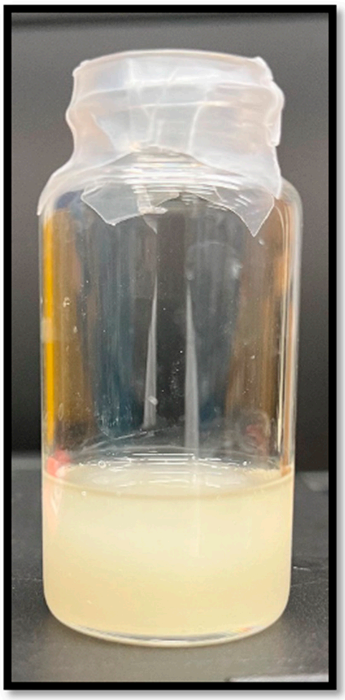

Figure S1. The mixture of the PSf and PVDF-HFP dope solutions resulted in a cloudy appearance, indicating phase separation and immiscibility due to low affinity.
